# Sodium-dependent glucose transporter 2 inhibitor alleviates renal lipid deposition and improves renal oxygenation levels in newly diagnosed type 2 diabetes mellitus patients: a randomized controlled trial

**DOI:** 10.1186/s13098-023-01236-1

**Published:** 2023-12-07

**Authors:** Li Zhang, Tongdan Wang, Yan Kong, Haizhen Sun, Yuling Zhang, Junmei Wang, Zhida Wang, Shan Lu, Pei Yu, Saijun Zhou

**Affiliations:** NHC Key Laboratory of Hormones and Development, Tianjin Key Laboratory of Metabolic Diseases, Chu Hsien-I Memorial Hospital & Tianjin Institute of Endocrinology, Tianjin Medical University, Beichen District, No.6 North Huanrui Rd, Tianjin, 300134 China

**Keywords:** Canagliflozin, Type 2 diabetes, Intrarenal lipid content, Renal oxygenation level, Hypoxia

## Abstract

**Background:**

Sodium-dependent glucose transporter 2 inhibitor (SGLT2i) has the advantages of effectively lowering blood glucose levels and improving renal outcomes in diabetic patients. This study evaluated the effect of canagliflozin on intrarenal lipid content and oxygenation in newly diagnosed type 2 diabetes mellitus (T2DM) patients.

**Methods:**

A total of 64 newly diagnosed T2DM patients with normal renal function were randomly divided into canagliflozin (n = 33) and glimepiride control (n = 31) groups. All patients underwent functional magnetic resonance imaging (fMRI) scanning to assay patients' intrarenal lipid content and oxygenation level before and after 24 weeks of treatment. Furthermore, the relationship between body mass index and intrarenal lipid content in T2DM patients was analyzed and the correlation between changes in intrarenal lipid content and improvements in renal hypoxia was further assessed.

**Results:**

The canagliflozin group had a greater decrease in body weight and blood uric acid level than the glimepiride group (all P < 0.05). The intrarenal lipid content could be significantly reduced after canagliflozin treatment for 24 weeks. The R2* values, a parameter for quantifying the oxygen content in tissues and is inversely related to the oxygen content, of the renal cortex and medulla in the canagliflozin group decreased from the baseline by 6.40% (*P* < 0.01) and 12.09% (*P* = 0.000007), respectively. In addition, the degree of reduction of fat fraction (ΔFF) in the kidneys of the canagliflozin group was correlated with the degree of improvement of oxygenation level (ΔR2*) in the renal cortex (r = 0.422, *P* = 0.014).

**Conclusions:**

The early renal protective effect of SGLT2i in newly diagnosed T2DM patients may be partly attributed to the amelioration of renal hypoxia via the alleviation of ectopic lipid deposition in the kidneys.

*Trial Registration:* Chu Hsien-I Memorial Hospital of Tianjin Medical University (ChiCTR2000037951).

## Introduction

Type 2 diabetes mellitus (T2DM) has become a serious threat to human health worldwide. T2DM could cause macrovascular and/or microvascular pathologic changes, which increases the risk of myocardial infarction, heart failure, stroke and renal failure, and reduces survival [1]. Diabetic kidney disease (DKD) is one of the most common and serious diabetes microvascular complications and is also the primary cause of end-stage renal failure [2, 3]. The prevalence of diabetes in China is 12.8% and is continuing to rise [4]. Nearly 30%–40% of individuals with diabetes experience kidney failure, cardiovascular disease, and premature death [5, 6]. The early stage of diabetic nephropathy is a key stage that can be prevented and controlled, and early treatment can also reverse or reduce the occurrence of diabetic nephropathy and even end-stage nephropathy. However, the traditional clinical indicators, such as urinary albumin (UA) and serum creatinine (Scr), are not sensitive enough to support early diagnosis and treatment for diabetic nephropathy. Unfortunately, once patients enter a stage of clinical albuminuria, the progression of DKD and the occurrence of chronic renal failure cannot be completely prevented even with strict control of blood glucose and blood pressure, and adequate application of RAS blockers [3]. Therefore, how to identify and prevent the occurrence of diabetic nephropathy in the early stage remains a difficult problem to be solved.

Ectopic deposition of lipids in the kidneys can occur in the early stage of T2DM, and the accumulation of intrarenal lipids can cause damage to intrinsic renal cells, including mesangial cells, podocytes, and renal tubular cells, leading to the progression of DKD [7]. Moreover, the ectopic deposition of lipids in the kidneys is an important cause of hypoxia, and chronic hypoxia in the kidneys is also a common pathogenic pathway of kidney injury mediated by various pathogenic factors of diabetes [8]. Therefore, how to reverse the ectopic deposition of lipids in the kidneys and improve the hypoxia state of the kidneys in an early stage is of great importance for T2DM patients.

Recently, functional magnetic resonance imaging (fMRI) has become a powerful tool for noninvasive evaluation of early changes in renal function. Studies have indicated that kidney fMRI changes, such as ectopic adipose deposition and cortical and medullary oxygenation, can occur during the early stages of diabetic nephropathy [9]. Sodium-dependent glucose transporter 2 (SGLT2) inhibitors are antihyperglycemic drugs of a cardiorenal risk-reducing agent, which exert multiple metabolic benefits, including reduced body weight, reduced systolic and diastolic blood pressure, and reduced serum uric acid, independent of insulin secretion [10]. The CREDENCE study confirmed that the SGLT-2 inhibitor, canagliflozin, carries the advantages of effective hypoglycemia and improved renal outcomes in DM patients with chronic kidney disease (CKD) [11, 12], which has led it to be recommended by several authoritative guidelines as the optimal hypoglycemic scheme for diabetes patients with CKD, highlighting the position of SGLT-2 inhibitors in secondary prevention of diabetic nephropathy. The secondary endpoint and exploratory analysis of the study by Cavas et al. indicated that SGLT-2 inhibitors can reduce the risk of new-onset nephropathy [13], suggesting that such drugs have a promising application to the primary prevention of nephropathy; however, direct evidence remains lacking. Our previous study of a small sample of newly diagnosed diabetic patients confirmed that canagliflozin could alleviate renal hypoxia independently of changes in renal blood perfusion [14]. Both Dixon MRI and blood oxygenation level–dependent (BOLD) MRI are promising methods that are safe, accurate and reproducible [15–17]. Therefore, in this study, Dixon MRI and BOLD MRI were used to assess newly diagnosed diabetes patients from a larger sample size. We further explored whether canagliflozin could ameliorate renal hypoxia by alleviating renal ectopic lipid deposition, thus taking on an early renal-protection role.

## Materials and methods

### Subjects

Based on our previous study, canagliflozin improved renal cortical and medullary oxygen levels by 22.3% and 29.2%, respectively [14]. For a power of 90% and type I error of 2.5%, 19 patients were required for each group (*δ* =  − 0.15). According to the 1999 World Health Organization diabetes diagnostic criteria, 66 newly diagnosed T2DM patients were enrolled between August 2020 and April 2023 from Chu Hsien-I Memorial Hospital, Tianjin Medical University (Tianjin, China), and 33 patients in the canagliflozin group and 31 patients in the glimepiride group completed the trial. Patients were divided into two groups using stratified blocking randomization according to body mass index (BMI) levels.

Inclusion criteria included patients (1) who were older than 18 years of age and newly diagnosed T2DM (2) whose glycosylated hemoglobin (HbA1c) was 7.5%–10.5% in the early screening and 7.0%–10.5% before the visit; (3) whose fasting blood sugar was less than 13.3 mmol/L; (4) whose body mass index (BMI) was between 18.5–45.0 kg/m^2^; and (5) whose fasting C-peptide level was more than 0.33 nmol/L. Exclusion criteria were (1) pregnant or planning to become pregnant, breastfeeding, or had the desire to give birth in the near future; (2) acute diabetic complications, such as diabetic ketoacidosis, hyperosmotic hyperglycemia syndrome, lactic acidosis or hypoglycemic coma; (3) poor blood sugar control who must use insulin; (4) chronic liver disease; (5) acute cardiovascular disease; (6) urine ACR > 30 mg/g; (7) glomerular filtration rate < 90 mL/min/1.73 m^2^; (8) severe infection within 6 months; (9) prior or current treatment with SGLT-2 inhibitor therapy; (10) allergic to SGLT-2 inhibitors or glimepiride; (11) suffering from poorly controlled hypertension; (12) previous gastrointestinal surgical history; (13) previous history of immune dysfunction or malignant tumor; (14) using glucocorticoids or other drugs that may affect blood sugar; (15) using loop diuretics; (16) implanted with a cardiac pacemaker, nerve stimulator, or artificial metal heart valve; (17) arterial aneurysm; (18) implanted with metal prostheses in the body; (19) severely high fever; (20) epilepsy, claustrophobia, or unable to cooperate with nuclear magnetic examination; and (21) unable to participate in the research process.

This study was registered in the online Chinese Clinical Trial Registry under the identifier ChiCTR2000037951 and was approved by the ethics committee of Chu Hsien-I Memorial Hospital of Tianjin Medical University (Tianjin, China). All patients provided their written informed consent prior to enrollment in the study.

## Methods

### Treatment

**Fig. 1:**
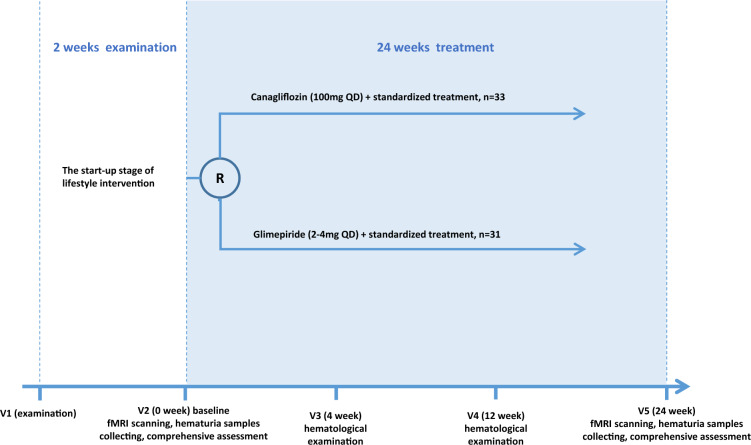
Flow diagram for study participants

This study was a prospective, randomized, and drug-controlled trial. All patients received two weeks of lifestyle intervention, including diet control and exercise guidance, before enrollment. During the observation period, four face-to-face visits were conducted (Fig. 1). Lifestyle guidance, drug dosage adjustments of standardized treatment, treatment of adverse events, and laboratory examinations were carried out at each visit. At V2 (Week 0) and V5 (Week 24), HbA1c, liver and kidney function, serum lipid concentrations, MRI, and other examinations and a comprehensive system evaluation were carried out. At V3 and V4, hematological examinations were conducted.

The experimental group received treatment with canagliflozin plus standardized treatment and the control group received glimepiride plus standardized treatment (standardized treatment = lifestyle intervention ± metformin). The target blood glucose was as follows: fasting blood glucose (FBG) 4.4–6.1 mmol/L and P2BG 5.6–7.8 mmol/L. The dose of metformin can be adjusted at V3 or V4 based on the blood glucose (0.5–2 g/day). The experimental group was given 100 mg canagliflozin daily before breakfast and continued with therapy until the end of the course. The control group took 2–4 mg glimepiride before breakfast, and the dosage of glimepiride and metformin was adjusted according to the blood glucose level.

### Data collection and laboratory examination (secondary indicator)

We collected clinical data, including age, sex, duration of diabetes, and drug allergy history. The weight, height, and brachial artery blood pressure of the right upper limb of each patient were measured by trained personnel before and 24 weeks after medication administration. Fasting venous blood and morning urine were collected and tested at the central laboratory of Chu Hsien-I Memorial Hospital, Tianjin Medical University. A Beckman Coulter AU5800 automatic biochemical analyzer (Beckman Coulter, Brea, CA, USA) was used to detect glycosylated serum albumin, liver and kidney function, blood lipid concentrations, and other biochemical indices. Patients’ HbA1c levels were tested using TOSOH G8 (Tosoh Bioscience, Tokyo, Japan). The laboratory team was not aware of how patients were grouped.

### MRI scanning (primary indicator)

All patients were subjected to renal MRI scanning by trained personnel before and 24 weeks after medication. The Ingenia 3.0 Tesla superconducting MRI scanner with a 32-channel phased array coil (Philips, Amsterdam, Netherlands) was adopted. Fasting and water abstention for three hours before examination was required to unify the hydration status of the subjects' kidneys. Before scanning, subjects were given respiratory training. Patients were positioned in the supine position, head first, hands at the sides of the body, with breathing gating in the maximum range of abdominal breathing movement, and fixed with abdominal straps. All patients underwent routine T1WI, T2WI, Dixon MRI, and BOLD MRI examinations. BOLD MRI uses the fact that the magnetic properties of hemoglobin depend on its oxygenated state [15]. Its quantitative index R2* values are directly proportional to the content of deoxyhemoglobin and inversely proportional to the partial pressure of oxygen. Dixon technique [16, 17], which is based on the chemical shift between water and fat signals, inphase and opposed-phase, and subsequent fat and water images are generated, from which the proton density fat fraction can be calculated as the fraction of fat signal in the fat and water signal in each voxel. Both Dixon MRI and BOLD MRI are promising methods that are safe, accurate and reproducible. The MRI radiologists were not aware of how the patients were grouped.

### Statistical analysis

Statistical analysis was conducted using IBM SPSS version 25.0 (IBM Corp., Armonk, NY, USA). A *t*-test was used to compare data groups with a normal distribution, while a chi-squared test was used to compare data groups with a non-normal distribution. *P* < 0.05 was considered statistically significant.

## Results

### Baseline clinical data of two groups of newly diagnosed T2DM patients

No significant statistical differences were found with regard to age, sex, BMI, weight, blood lipid, SBP, DBP, FBG, HbA1c, liver and kidney functions, and other baseline clinical data between the two groups of patients with newly diagnosed T2DM with normal renal function (Table 1).

**Table 1:** Comparison of two groups baseline clinical data and changes in metabolic data recorded after treatment between two groups of newly diagnosed T2DM patients

	Glimepiride group (n = 31)	Canagliflozin group (n = 33)	P value
Age	47.52 ± 11.72	47.18 ± 11.54	0.909
Sex (M/F)	19/12	17/16	0.431
BMI (kg/m^2^)	26.12 ± 3.14	26.70 ± 3.06	0.455
Weight (kg)	76.53 ± 15.21	77.52 ± 12.79	0.779
FBG (mmol/L)	8.31 ± 2.11	7.69 ± 1.54	0.187
HbA1C(%)	8.54 ± 1.50	8.14 ± 1.42	0.279
CHO-L (mmol/L)	5.10 ± 1.69	5.13 ± 1.66	0.942
LDL-C (mmol/L)	3.39 ± 0.95	3.32 ± 0.81	0.753
TG (mmol/L)	2.07 ± 1.00	2.16 ± 1.17	0.758
UA (umol/L)	339.70 ± 89.74	340.70 ± 92.13	0.967
Cr (umol/L)	62.87 ± 16.49	60.19 ± 12.63	0.466
BUN (umol/L)	6.76 ± 2.89	5.68 ± 1.59	0.065
ALT (IU/L)	23.51 ± 9.29	24.86 ± 11.37	0.605
AST (IU/L)	23.12 ± 8.72	22.79 ± 10.05	0.891
SBP (mmHg)	125.20 ± 9.28	128.80 ± 15.43	0.263
DBP (mmHg)	79.00 ± 8.79	81.33 ± 11.30	0.362
UACR (mg/g)	10.84 ± 5.87	12.26 ± 4.78	0.292
eGFR (ml/(min.1.73m^2^))	109.60 ± 17.03	110.90 ± 15.14	0.732

Data are presented as mean ± SD or median (interquartile range) values, p < 0.05

*BMI* body mass index, *FBG* fasting blood glucose *HbA1c* glycosylated haemoglobin *CHO-L *cholesterol *LDL-C* low density lipoprotein-cholesterol, *TG* triglyceride, *UA* uric acid, *Cr* Serum creatinine *BUN* blood urea nitrogen *ALT* alanine aminotransferase *AST* aspartate aminotransferase, *SBP* systolic blood pressure, *DBP* diastolic blood pressure, *UACR* urinary albumin-to-creatinine ratio calculated with albumin measured in milligrams and creatinine measured in grams; Estimated glomerular filtration rate(eGFR) calculated according to the CKD-EPI formula

### Changes in metabolic data recorded after drug treatment compared to baseline

**Fig. 2:**
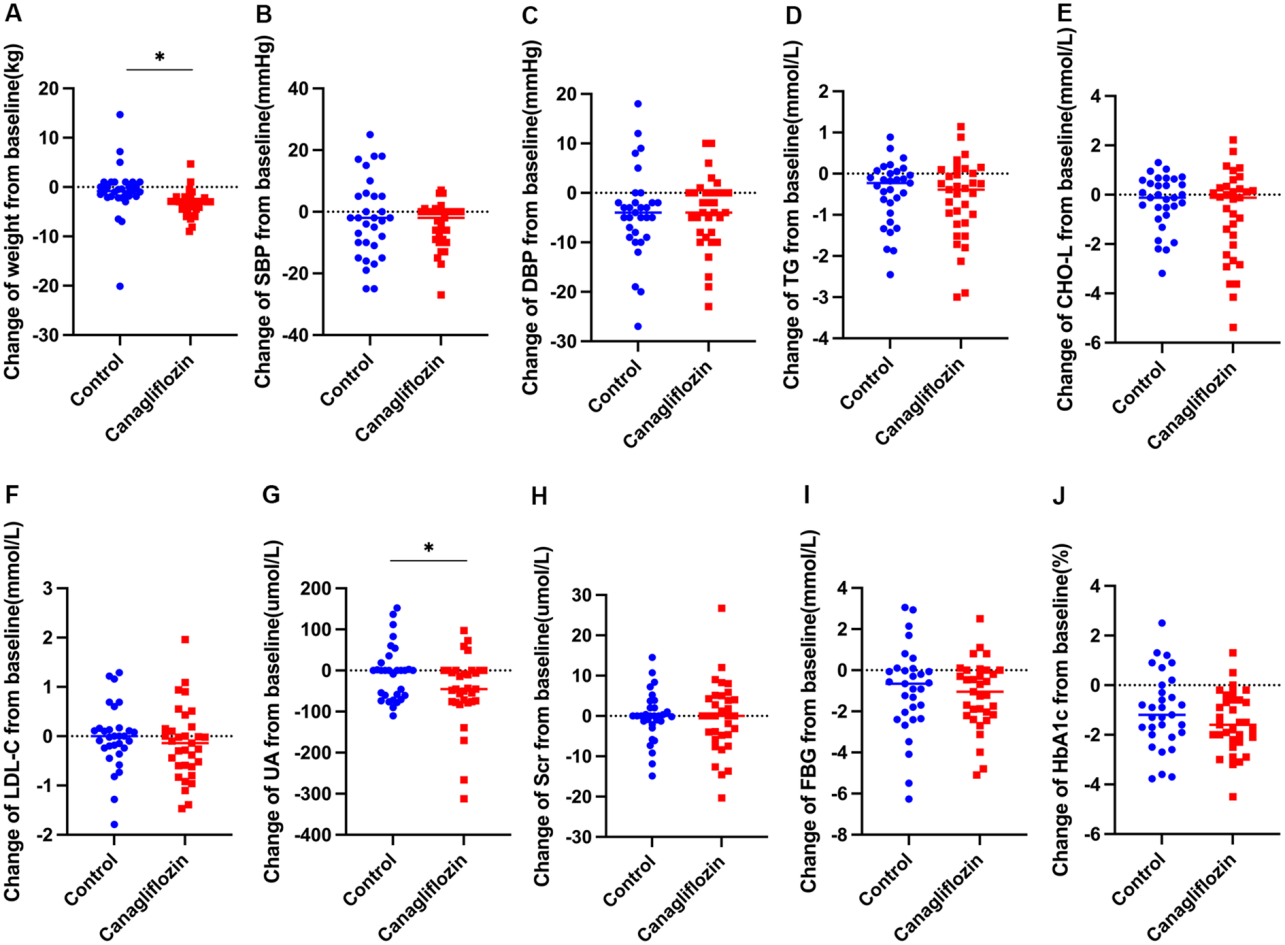
The improvement of metabolic data in two groups of newly diagnosed T2DM patients after treatment. **A** Weight **B** Systolic blood pressure (SBP) **C** Diastolic blood pressure (DBP) **D** Triglyceride (TG) **E** Total cholesterol (CHO-L) **F** Low density lipoprotein (LDL) **G** uric acid (UA) H. Serum creatinine (Scr) **I** Fasting blood glucose (FBG) **J** glycosylated hemoglobin (HbA1c) * P < 0.05,** P < 0.01

After 24 weeks of treatment, body weight and UA significantly decreased compared with those at baseline (Fig. 2A, G). As shown in Fig. 2G, the UA level of newly diagnosed T2DM patients can be significantly reduced by canagliflozin therapy (− 6.56 ± 66.25 vs. − 46.49 ± 83.75 μmol/L, *P* = 0.04), and this effect may be independent of the hypoglycemic effect and lifestyle. After 24 weeks of treatment, the body weight of the canagliflozin group decreased by 3.00 (2.55, 4.50) kg from the baseline, which was a greater reduction than that of the glimepiride control group (0.8 (− 1, 1.90) kg, *P* < 0.05) (Fig. 2A). There was no significant difference in total cholesterol, low-density lipoprotein cholesterol, triglyceride levels, FBG and HbA1c between the two groups from the baseline.

### Changes in renal fat content in two groups of newly diagnosed T2DM patients before and after treatment

**Fig. 3:**
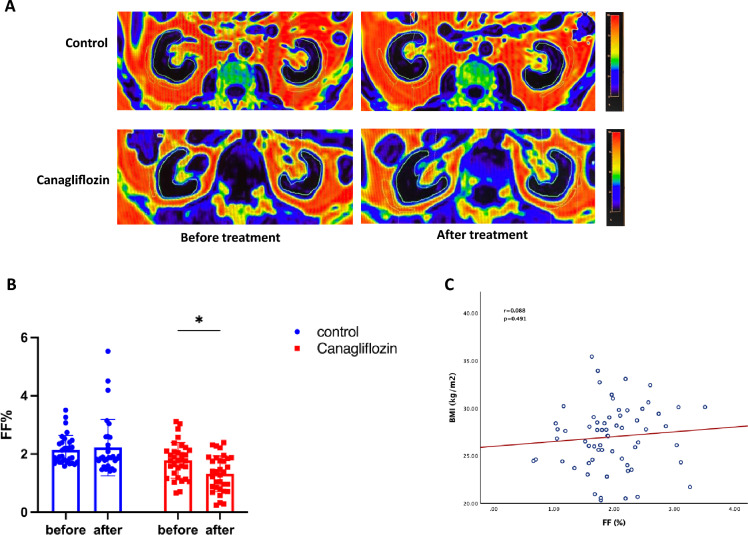
Effect of Canagliflozin on renal fat fraction (FF) in newly diagnosed T2DM patients. **A** Kidney Dixion MRI images of two groups of newly diagnosed patients before and after treatment **B** Changes of renal FF in two groups before and after intervention **C** Analysis of correlation between baseline BMI and renal FF. * P < 0.05,** P < 0.01

The Dixon MRI technique is a noninvasive and nonradioactive method for quantitative detection of fat content. The results showed that there was no statistical difference in baseline renal fat fraction (FF) between the two groups of newly diagnosed T2DM patients. After treatment, the renal FF of the canagliflozin group decreased significantly (1.79 ± 0.61 vs 1.32 ± 0.62, *P* = 0.0445) (Fig. 3B), which decreased by 0.47% compared with that at baseline. We further analyzed the correlation between baseline BMI and renal FF, as shown in Fig. 3C. There was no significant correlation between renal FF and BMI in this group of T2DM patients (r = 0.088, *P* = 0.491), which indicated that BMI does not reflect the degree of ectopic fat deposition in T2DM patients. Canagliflozin can significantly reduce renal fat accumulation.

### Changes in renal cortical and medullary oxygenation levels before and after treatment

**Fig. 4:**
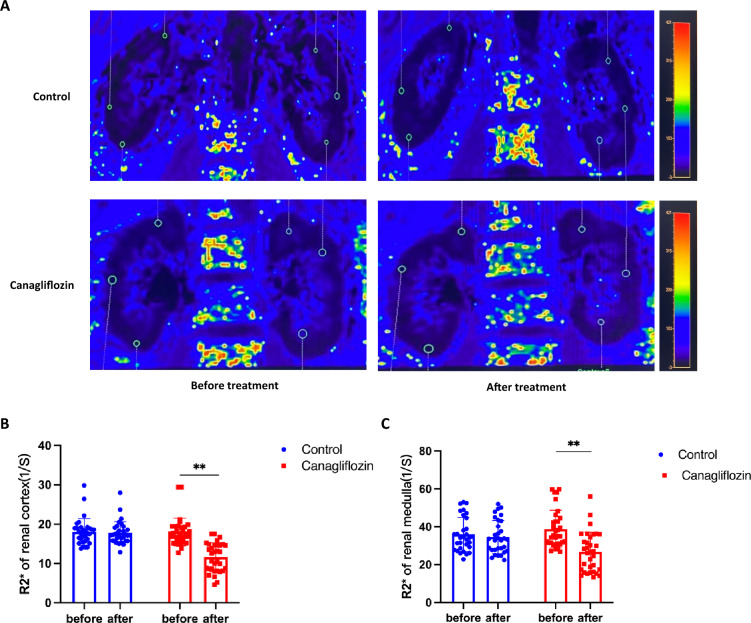
Changes of renal cortex and medulla oxygenation level before and after treatment in two groups. **A**Kidney BOLD MRI images of two groups of newly diagnosed patients before and after treatment (“○” in figure represents the region of interest) **B** The change of R2* value of renal cortex in two groups before and after treatment **C** The change of R2* value of renal medulla in two groups before and after treatment. * P < 0.05,** P < 0.01

Based on the evidence that canagliflozin can significantly reduce the accumulation of fat in the kidneys of newly diagnosed T2DM patients, the changes in renal hypoxia were further analyzed. BOLD MRI imaging of the kidneys is a noninvasive detection method with high sensitivity to the changes in the kidneys’ oxygenation state. Its evaluation parameter is R2*, a parameter for quantifying the oxygen content in tissues, which is inversely related to the oxygen content. As shown in Fig. 4B, the baseline R2* values of the bilateral renal cortices in the canagliflozin group and control groups were 17.98 ± 3.54 and 18.05 ± 3.41 1/s, which decreased to 11.58 ± 3.66 and 17.77 ± 2.89 1/s after 24 weeks of treatment, respectively. The oxygenation parameter R2* value of the renal cortex in the canagliflozin group decreased by 6.40% from the baseline (*P* < 0.01), while the control group decreased by 0.28% from the baseline but without a statistical difference (*P* = 0.99). The R2* values of the renal medulla at the baseline in the two groups were 38.79 ± 9.99 and 35.98 ± 8.92 1/s, which decreased to 26.70 ± 10.45 and 34.62 ± 8.77 1/s after 24 weeks of treatment, respectively. The R2* value of the renal medulla in the canagliflozin group decreased by 12.09% from the baseline (*P* < 0.01), and that in the control group decreased by 1.36%, also without reaching a statistical difference (*P* = 0.94). Canagliflozin can improve renal cortex hypoxia.

### Correlation between the reduction in renal lipid deposition and the improvement of renal cortex and medulla oxygenation levels

**Fig. 5:**
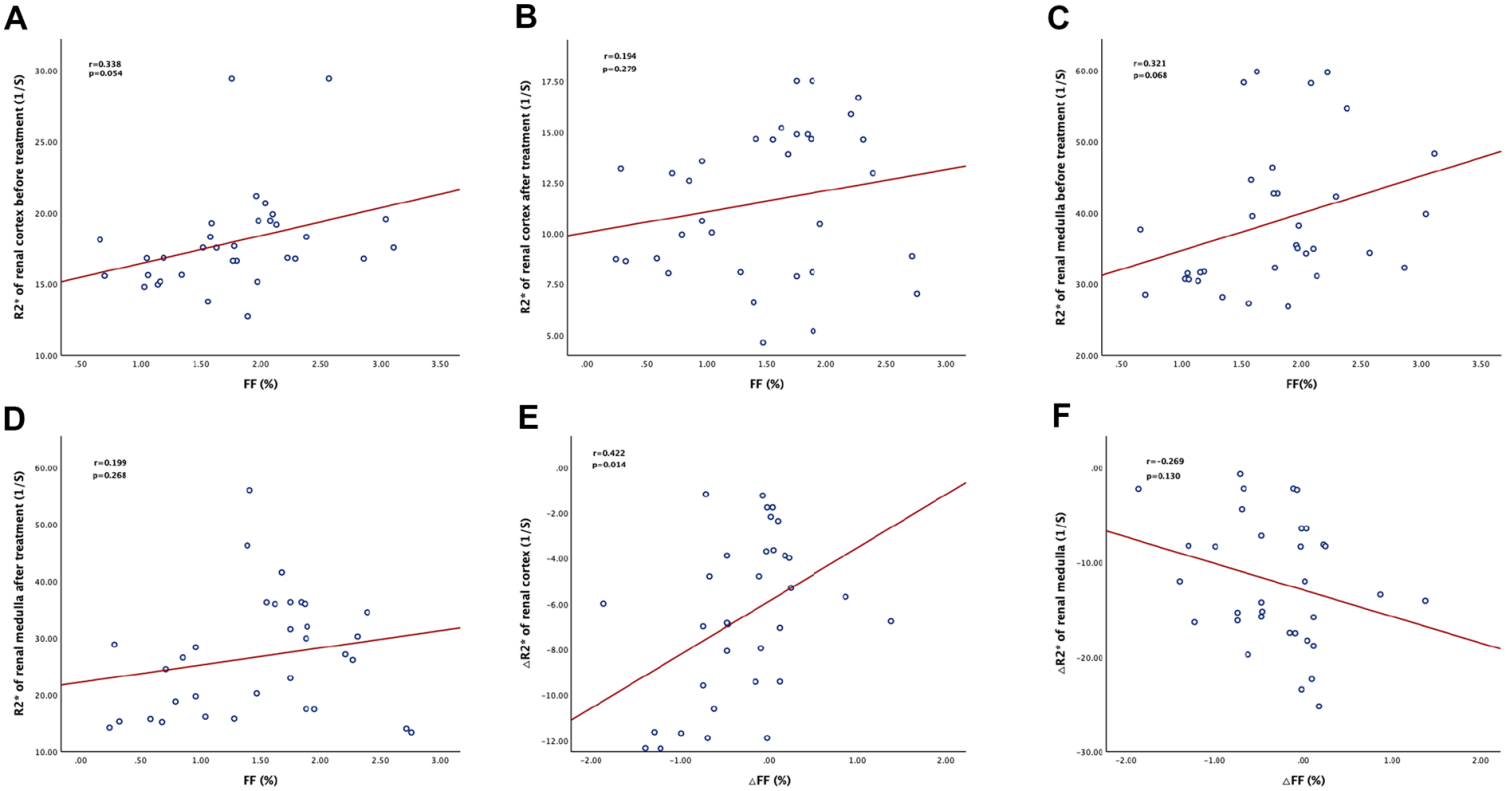
Correlation between the improvement of renal oxygenation level and the change of renal fat deposition in newly diagnosed type 2 diabetic patients after treatment with Canagliflozin. **A**, **B**. Correlation analysis of renal cortex R2* value and renal fat fraction (FF) before and after treatment **C**, **D** Correlation analysis of renal medulla R2* value and renal FF before and after treatment **E**, **F**. Correlation analysis of renal cortex and medulla △R2* value and △FF after treatment

We also analyzed the correlation between the renal FF with renal cortex R2* and medulla R2* in T2DM patients at the baseline and after treatment with canagliflozin. As shown in Fig. 5A–D, there was no significant correlation between renal FF and the renal cortex R2* or medulla R2* in T2DM patients at the baseline and after treatment with canagliflozin. In this study, we further analyzed the correlation between the reduction in renal fat content and the improvement of renal cortex and medulla hypoxia in the canagliflozin group. As shown in Fig. 5E and F, the change in renal FF (△FF) was significantly correlated with the change in renal cortex R2* (△R2*) (r = 0.422, *P* = 0.014), while the correlation between renal △FF and medulla △R2* did not show a statistical difference.

## Discussion

The degree of heterotopic lipid deposition in T2DM patients is much higher than that in non-diabetic patients; this not only promotes insulin resistance, impairs pancreatic β-cell function, and aggravates metabolic disorder but also is an important pathological basis leading to the damage of target organ function. The accumulation of lipids in organs can lead to local lipotoxic injury. To date, a large number of studies have focused on the ectopic deposition of fat in the liver and pancreas, leading to the formation and progression of nonalcoholic fatty liver and promoting the damage of pancreatic β-cells. However, there is little research on renal lipid ectopic deposition. Some studies have confirmed that the accumulation of fat in the kidneys is also an independent risk factor for kidney injury. Therefore, reversing or reducing ectopic lipid deposition in the kidneys early on is an effective strategy for early kidney protection in patients with T2DM. A large pool of data from evidence-based medicine has proved that SGLT2 inhibitors are antihyperglycemic drugs of a cardiorenal risk-reducing agent [18, 19]. Previous studies have concluded the potential mechanisms that contribute to their protective effects on the kidney include (1) restoration of the tubuloglomerular feedback, (2) decreased activation of the intra-renal renin–angiotensin–aldosterone system, (3) increased production of ketone bodies, and (4) protection against oxidative stress, and fibrosis [20]. In addition, studies have confirmed that SGLT-2 inhibitors can lessen abdominal visceral fat content and liver fat accumulation in T2DM patients while simultaneously reducing their weight [21, 22]. These studies suggested that SGLT-2 inhibitors can display a renal-protection mechanism independent of its hypoglycemic, hypotensive, and tubuloglomerular feedback by reducing renal fat content. In this study, the findings suggested that weight loss can be achieved through short-term lifestyle intervention, but the extent to which fat content in the kidneys is reduced is limited. However, canagliflozin can significantly reduce the content of fat in the kidneys.

The accumulation of fat in the kidneys can not only lead to lipotoxic injury but also exacerbate renal hypoxia. Chronic renal hypoxia is also a common pathogenic pathway of kidney injury mediated by various pathogenic factors of diabetes, which is an important trigger that induces and aggravates oxidative stress injury [23]. BOLD MRI is currently the only noninvasive technique that can detect the oxygenation level of tissues in vivo, which can reflect the oxygen content and oxygen saturation of tissues so as to reflect the functional state of tissues, and it shows good consistency with the oxygen partial pressure measured directly by microelectrodes [24–26]. The kidney is a highly perfused organ, and the cortex–medulla pairing has a natural oxygen partial pressure gradient, making it an ideal organ for BOLD MRI examination. BOLD MRI can use deoxyhemoglobin as an endogenous contrast agent to indirectly reflect the partial pressure of local tissue oxygen without increasing the metabolic burden on the kidneys, evaluate the blood oxygen level of the tissue, and make it possible to reflect kidney function earlier than the morphological changes of the kidney [25, 26]. The measurement results of the BOLD MRI parameter R2* have high reliability. The results showed that canagliflozin can improve hypoxia of the renal cortex and medulla in newly diagnosed diabetic patients, which may confirm a protective effect of canagliflozin on the kidneys independent of lowering blood glucose. This phenomenon may be related to the mechanism that SGLT2 inhibitors could induce an overall metabolic shift toward a fasting state, characterized by the increased use of lipids and ketones as energy substrates, which may improve oxygen availability and attenuate renal hypoxia [27, 28]. Previous study also discovered that SGLT2 inhibitors could reduce HIF-1 activity and/or promote HIF-2α activity, increasing erythropoietin [29]. The latter increases hematocrit and improves O_2_ delivery to the kidney medulla and cortex [29]. This effect may be related to SGLT2 inhibitors’ ability to alleviate renal glycolipid toxicity, thereby reducing the inflammatory response. The results also suggested that canagliflozin may have a primary preventive effect against diabetic nephropathy.

Based on the aforementioned findings, the correlation between the reduction in renal FF and the improvement in hypoxia was further analyzed. A significant correlation between the decrease in renal FF and the oxygenation level of the renal cortex was found. These results suggested that the improvement of the renal cortex oxygenation level brought on by canagliflozin related to the reduction in renal fat deposition.

Moreover, canagliflozin can significantly improve renal medulla hypoxia, but the improvement in renal medulla oxygenation level showed no significant correlation with the reduction of renal FF. The reason for this trend may be that the R2* value of the renal medulla was higher than that of the renal cortex in T2DM patients with normal renal function in this study, whether at baseline or after treatment. Canagliflozin can improve renal cortex hypoxia by reducing the accumulation of renal fat, while the mechanism of renal medullary hypoxia and renal cortical hypoxia in T2DM is different. The renal medulla itself has a low oxygen content, but the renal tubule consumes much oxygen in the reabsorption process of macromolecular substances and ions. Such a physiological function of low oxygen and high load determines that the renal medulla is more susceptible to ischemia and hypoxia. Under the condition of glomerular hyperfiltration, the renal tubule reabsorption burden, sodium pump activity, and cell oxygen consumption all increased. Conversely, after treatment with canagliflozin, sodium uptake by renal tubules and oxygen consumption of cells decreased, thus alleviating hypoxia in the kidney medulla. Therefore, the improvement of renal medulla hypoxia caused by canagliflozin is related to the reduction of renal tubular reabsorption of sodium and glucose. Hesp et al. also speculated in their review that SGLT-2 inhibitors may play a protective role in the kidneys by improving hypoxia of the renal cortex in DKD patients [30]. Our research not only confirmed the hypothesis of Anne et al. in newly diagnosed diabetic patients but, also, the newly diagnosed T2DM patients enrolled in this study all had normal renal functions, so these patients were in diabetic CKD1 stage. The results also suggested that renal cortex and medullary hypoxia could occur in the early stage of DKD. Zanchi et al. did not observe the effect of empagliflozin on the oxygenation level of non-diabetic patients’ kidneys [31], which may be related to the fact that the kidneys of non-diabetic volunteers did not suffer from hypoxic damage. This study also confirmed the renal damage caused by chronic hypoxia in patients with T2DM from another perspective. Therefore, improving the hypoxia state of the kidneys early on is beneficial to inhibit the progression of DKD. Canagliflozin can protect the kidneys by improving the oxygenation level of newly diagnosed T2DM patients.

Moreover, in our study, only two patients experienced side effects of mild urinary tract infections, and four patients had mild urine ketone body positivity. We also did not observe any adverse reactions associated with interactions of canagliflozin and other drugs such as metformin. Canagliflozin performed a good safety profile in these newly diagnosed T2DM patients overall.

Limitations of this study include a relatively modest sample size, and this was a single-center study. The findings should be further verified in a larger, multicenter study, and the specific molecular mechanism needs further investigation. Meanwhile, long-term follow-up data are required to determine whether the observed beneficial effects of canagliflozin on renal outcomes are sustained over time.

To sum up, canagliflozin can reduce the ectopic deposition of renal lipids and improve chronic renal hypoxia in newly diagnosed T2DM patients with normal renal function. The use of SGLT-2 inhibitors during the early stages of T2DM may have an early renal benefit.

## Data Availability

The datasets used and/or analyzed during the current study are available from the corresponding author upon reasonable request.
